# Relation of Different Components of Climate with Human Pituitary-Thyroid Axis and FT3/FT4 Ratio: A Study on Euthyroid and SCH Subjects in Two Different Seasons

**DOI:** 10.1155/2019/2762978

**Published:** 2019-01-16

**Authors:** Taha O. Mahwi, Darya S. Abdulateef

**Affiliations:** ^1^Department of Medicine, College of Medicine, University of Sulaimani, Sulaymaniyah 46001, Iraq; ^2^Physiology Department, College of Medicine, University of Sulaimani, Sulaymaniyah 46001, Iraq

## Abstract

**Background:**

Various changes in thyroid hormones (TH) and thyroid-stimulating hormone (TSH) level were observed in different seasons among euthyroid and hypothyroid subjects living in areas with an extreme temperature difference between summer and winter.

**Objectives:**

This study aims at finding the effect of temperate climate on the seasonal variations of TSH and TH in euthyroid and subclinical hypothyroidism (SCH) subjects and at evaluating if the test season has an effect on the number of subjects diagnosed as SCH. It basically focuses on the relation of different components of climate with TH and TSH.

**Method:**

In a prospective study on 152 healthy (euthyroid) volunteers and 25 SCH subjects, the serum hormone levels (TSH, FT4, and FT3) were measured in both the summer and winter seasons and correlated with all the climate components using Pearson's correlation coefficient. The effect of duration of outdoor exposure on hormone levels was compared using a paired sample *t*-test (*P* < 0.05).

**Results:**

Small but statistically significant increased FT3 level and decreased FT4 level were observed during the winter season in euthyroid and SCH subjects, respectively. There was a significant negative correlation between FT3 and FT3/FT4 ratio with temperature and sunshine duration and a positive correlation with humidity and atmospheric pressure. A positive correlation was found between FT4 and sunshine duration.

**Conclusion:**

The climate components contributed to the slight variance in hormone levels in different seasons, and the effect was mostly on peripheral conversion of FT4 to FT3 rather than the pituitary-thyroid axis leading to slightly higher FT3 in winter. Seasonal variation does not affect the diagnosis of SCH cases.

## 1. Introduction

The hypothalamic-pituitary-thyroid (HPT) axis, also known as thyroid homeostasis, is a part of the neuroendocrine system, responsible for the biosynthesis and secretion of thyroid hormones (TH) thyroxine (T4) and triiodothyronine (T3). This is regulated by the release of thyroid-stimulating hormone (TSH) from the anterior pituitary, which in turn is stimulated by the release of thyrotropin releasing hormone (TRH) from the hypothalamus. The decrease in T4 and T3 is a stimulator for the release of TSH and TRH, operating in a process known as negative feedback [1]. Most of the serum T3 originates from the conversion of T4 by extrathyroidal type 2 iodothyronine deiodinase (D2) [2]. The thyroid hormones are responsible for the regulation of metabolism and cardiovascular system, development of the nervous system, linear growth, and thermogenesis [3, 4]. As these hormones facilitate adaptation to temperature variation, seasonal changes in TSH and/or TH levels have been observed among healthy (euthyroid) individuals exposed to extreme environmental conditions, as in the arctic and subarctic regions, and those with prolonged stay in Antarctica [5–10]. Most of the studies showed an increase in TSH in winter [6, 9] or with prolonged exposure to cold temperatures [7]; however, a few studies exhibited an increase in T3 and/or T4 [7, 10], while a decrease in the TH levels was also observed [6–8]. The cause of these seasonal changes in thyroid hormone and TSH level varies; it could be due to a centrally mediated response of the hypothalamic-pituitary-thyroid axis [8, 11], change in thyroidal secretion [12], change in protein binding [13], ambient luminosity [6], or peripheral metabolism of thyroid hormone during different seasons [14].

Circannual variations in the thyroid hormone levels have also been studied in regions having a temperate climate, where a vast difference in temperature exists between summer and winter [15–17]. Most of the studies lacked reliability as they were conducted before the development of new and efficient assays [11, 17, 18] or the sample size was small resulting in erroneous data analysis [15, 17] or it was a retrospective study [19]. The effects of higher temperatures on the hormones are still not known as earlier studies were focused in areas where the maximum temperature rarely exceeded 35°C.

Studies on the seasonal changes in the TSH levels in euthyroid and hypothyroid residents of temperate climates [15, 16, 20] have reported an increase in serum TSH and a decline of serum T4 during the winter month; in some cases, these changes may be clinically relevant [16]. A recent Korean study, held retrospectively, demonstrated significant seasonal differences in serum TSH levels which led to a temporary transition between the euthyroid status and subclinical hypothyroidism in individuals with borderline normal-high TSH [19]. In hypothyroid patients, the physiological adaptation of the thyroid to seasonal variations does not occur normally; the maintenance of TSH secretion is done by replacement therapy with L-thyroxine (L-T4), the dose depending on the seasonal variations in the basal and TRH-stimulated TSH [19]. Subclinical hypothyroidism (SCH), an insufficiently studied condition of the thyroid is identified by the mild elevation of serum TSH levels without obvious symptoms. The reversible nature of SCH along with the lack of predictors, except for detection of antithyroid peroxidase (TPO) antibodies, leads to difficulties in the management of the condition [19].

The absence of a thorough investigation on the relation of the pituitary-thyroid axis (PTA) with all components of climate and the duration of outdoor exposure and the variability in the results for the relation between TH and temperature and luminosity is a major drawback. In this study, we probed the seasonal variations in the levels of thyroid-stimulating hormone, thyroid hormones FT3 and FT4, and the FT3/FT4 ratio in euthyroid and SCH subjects from the city of Sulaymaniyah, Iraq, a latitude still unexplored, with a wide seasonal difference where the maximum temperature in summer exceeds 45°C and in winter it goes down to 0°C and sometimes as low as -5.6°C. The effect of climate components such as temperature, humidity, sunshine duration, cloud cover, and atmospheric pressure and the duration of outdoor exposure on the hormone levels were determined in a study population comprising euthyroid subjects and subjects with subclinical hypothyroidism.

## 2. Materials and Methods

### 2.1. Subjects

The study was initiated with 217 healthy volunteers and 32 subclinical hypothyroidism (SCH) subjects. Healthy volunteers included family, friends, healthy hospital employees, and their relatives and patient escort. The study was approved by the Ethical Committee of the College of Medicine/University of Sulaimani, and informed written consent was taken from all the participants. A detailed questionnaire was filled out for each patient. The questionnaire contained sociodemographic status (age, height, weight, waist circumference, and details about employment and lifestyle), history of thyroid and systemic diseases, drug intake, and surgery. It also included the degree of natural exposure to the outdoor atmosphere, based on which the subjects were divided into two groups: good exposure group (>3 h) and slight exposure group (<3 h). The exclusion criteria for subjects included (i) abnormal serum TSH levels (<0.5 mU/l or> 10 mU/l), (ii) abnormal free thyroid hormones T4 (FT4) or T3 (FT3), (iii) history of thyroid diseases, (iv) presence of thyroid nodule, (v) antithyroid drug treatment, (vi) chronic diseases such as diabetes, (vii) acute illness, (viii) hyperprolactinemia, and (ix) inpatients. During the summer season, the subjects selected based on the questionnaire underwent thyroid function tests and were investigated for the proportion of the antithyroid peroxidase antibody (TPO-Ab) in the serum. After baseline measurements, 19 subjects who had at least one of the exclusion criteria were eliminated from the study. Based on their serum TSH level, the subjects were separated into two groups: 40 subclinical hypothyroidism (SCH) and 190 euthyroid subjects. In the follow-up study in the winter season, total 53 subjects were excluded either due to the development of overt hypothyroidism and thyrotoxicosis, due to pregnancy, or as they did not turn up for the follow-up test. Among the 177 subjects remaining, 25 subjects with subclinical hypothyroidism and 152 subjects with euthyroidism were included in the final analysis.

### 2.2. Biochemical Measurements

Blood samples were collected from the participants between 9 : 00 and 11 : 00 A.M. after an overnight (12 h) fast. Blood was allowed to clot at room temperature and centrifuged to separate the serum. The serum was analyzed for FT3, FT4, and TSH and antibody titres against thyroid peroxidase (TPO). All the biochemical tests were performed by electrochemiluminescence immunoassay (ECLIA) and analyzed using the same device, the Cobase 411 analyzer (Roche Diagnostics, GmbH, Germany). The reference ranges, as determined in the laboratory for FT3, FT4, and TSH, were 2.0-4.4 pg/ml, 0.93-1.70 ng/dl, and 0.27-4.2 mIU/l, respectively. Thyroid peroxidase antibody (TPO-Ab) up to 34 IU/ml was considered normal.

Euthyroid was defined as a subject with TSH and FT4 within the reference range, subclinical hypothyroid (SCH) means elevated TSH (>4.2 mIU/l), with normal FT4 (determined after two tests taken two weeks apart), and thyroid peroxidase (TPO) positivity was defined as having TPO-Ab above 34 IU/ml.

### 2.3. Climatic Data

The detailed climatic data for each day during the study period was obtained from the Metrological Station of Sulaimani, Sulaymaniyah city. The mean of each climatic component of both the summer and winter seasons are demonstrated in Table 1.

### 2.4. Statistical Analysis

The data were analyzed statistically using IBM SPSS Statistics version 22 software (IBM Corporation, New York, USA). Descriptive statistics was performed; the demographic data, climate components, and hormone levels were represented as the mean ± SD. The mean of each climatic component and the hormone levels between the summer and winter seasons were compared using a paired *t*-test. The comparison between the slight and good exposure groups was done in each season using an independent sample *t*-test. *P* ≤ 0.05 was regarded as statistically significant. The correlation between variables was evaluated using Pearson's correlation coefficient, and step-wise regression analysis was performed for the prediction of significant correlations.

## 3. Results

### 3.1. Study Subjects

Table 2 gives the demographic details of the subjects in this study. After the exclusion of the subjects either due to their failure in returning for the follow-up or due to the development of thyroid diseases or pregnancy, the number of study subjects entering for the analysis was 177.

### 3.2. Climatic Components of Seasonal Variations

The mean of each climatic component has been demonstrated in Table 1. A statistically significant difference was observed (*P* < 0.001) between the measured components during the summer and winter seasons.

**Figure 1:**
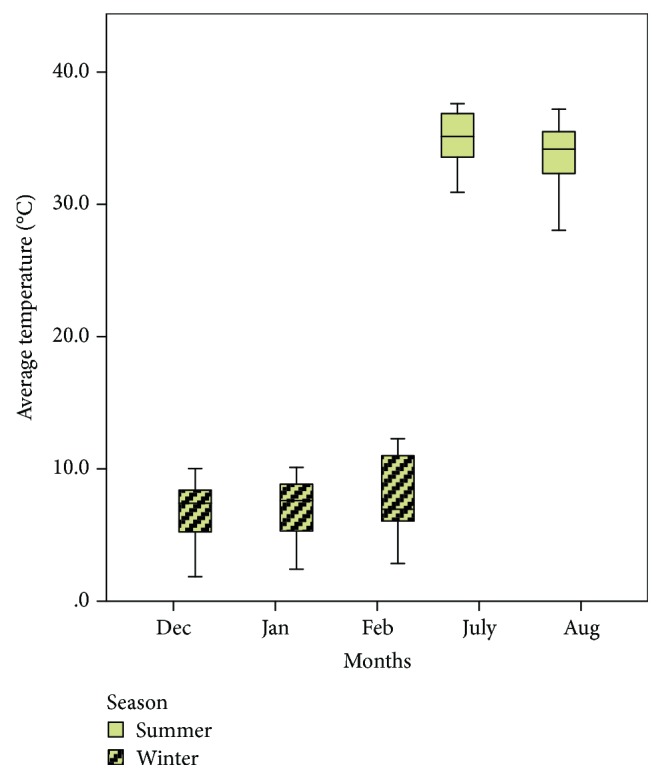
The boxplots of average temperature, during each month of sample taking in summer and winter seasons.

In Figure 1, the average temperature means of different months were demonstrated throughout summer and winter seasons. There were no significant differences in average temperature during different months of the same season (*P* ≤ 0.05).

### 3.3. Thyroid Hormone Measurements

The variations in the levels of the thyroid hormones are presented in Table 3. The level of FT3 was found to be higher in winter whereas the FT4 level was high during summer with the statistical significance observed only for euthyroid and SCH subjects, respectively. The ratio of FT3/FT4 was higher during winter in both the study groups and was statistically significant compared to summer. The comparison of subjects on the basis of duration of outdoor exposure revealed an increase in FT3 during winter in subjects with good exposure (*P* = 0.023), with no significant differences between other variables as illustrated in Table 4.

**Figure 2:**
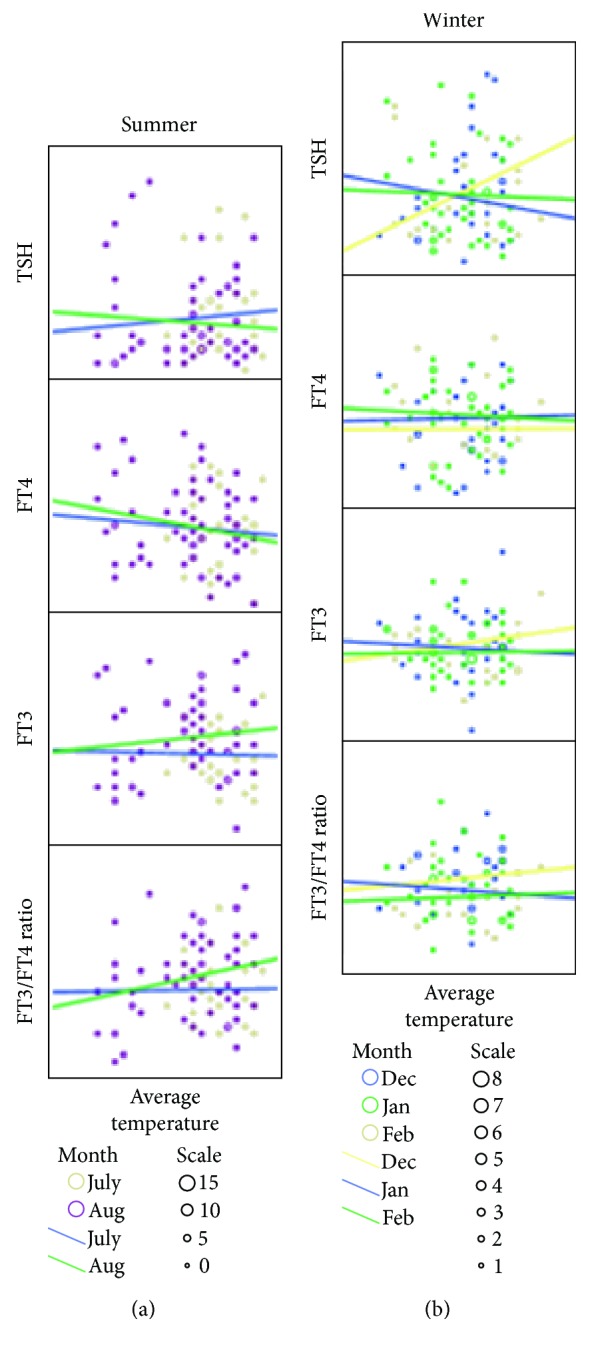
Changes of TSH, FT3, FT4, and FT3/FT4 ratio, according to the monthly temperature within the seasons. The scatter plots in both summer (a) and winter (b) are shown.

The relation between average temperature and thyroid function in different months of blood sample taking is demonstrated in Figure 2. The data of both the summer and winter seasons were shown in a scatterplot matrix.

Correlation analysis between the different climate components measured one day, one week, and one month before blood sample collection and the hormone levels was carried out using Pearson's correlation analysis as shown in Table 5. FT3 showed a negative correlation with temperature while a positive correlation was observed with humidity and atmospheric pressure. FT4 showed a negative correlation (*r* = −0.136) with the cloud cover, tested a day before. There were significant correlations between climate components of the day before blood testing and FT3 and climate components of the day and month before testing with the FT3/FT4 ratio. Temperature and sunshine duration were negatively correlated while humidity and atmospheric pressure were positively correlated to FT3 (*P* < 0.05). Positive correlation was observed between the cloud cover and FT3/FT4 ratio, though it was negatively correlated to FT4 (*P* < 0.05). In the SCH group, no significant correlation was observed between climate components and FT3 level except for the negative correlation between TSH and the cloud cover (*r* = −0.0332, *P* = 0.0069) and FT4 and humidity (*r* = −0.247, *P* = 0.040), a day and a week earlier, respectively.

With regression analysis, 3.9% of the variance in FT3 was explained by the duration of sunshine a day prior to sampling, 5.1% by the humidity before one week, and 2.9% by the atmospheric pressure before one month of sampling. The variance in the FT3/FT4 ratio was 5.7% due to the temperature of the week before and 4.1% and 1% due to the duration of sunshine, a month and a day before sampling, respectively. In the SCH group, 6.1% variance in FT4 was due to humidity.

There were no significant correlations between hormone levels in SCH subjects. In euthyroid subjects, there was a positive correlation between and FT4 and FT3 (*r* = 213, *P* = 0.008), while a negative correlation existed between TSH and FT4 (*r* = −0.205, *P* = 0.001).

## 4. Discussion

Earlier studies on the effect of seasonal variations on humans were mostly performed in the polar region with the subjects exposed to freezing temperature during the winter season; even the temperature during summer was low compared to the summer temperature of the temperate climate. Prolonged residence of euthyroid individuals in an extremely cold climate (Antarctic residence) corresponded with increased serum TSH, decreased FT4, and increased T3 production with clearance [7, 21]. This adaptation to the cold climate developed after residing for more than five months. Notably, exposure to the sub-Antarctic region only increased T3, without any effect on the other thyroid hormones [21]. The effect of photoperiod on the thyroid hormone levels during summer and winter had been observed in the people residing in the Antarctica. There was a significant decrease in the TSH levels, while the FT3 level was significantly increased in winter, an observation upon exposure of individual to bright light [22]. The responses of serum FT4 and TSH at high altitudes have been discordant, supporting the concept that the hypothalamic-pituitary feedback of T4 may be altered near 5400 m. At altitudes of 5400 m and 6300 m, a progressive elevation in the levels of T3 and T4 was observed; interestingly, serum TSH levels were also high, indicating altered regulation of TSH secretion [23]. Another study at extreme altitudes also reported a similar result but showed a decrease in FT3 and FT4 at subzero temperatures [24], whereas the hormone responses after a Himalayan expedition were characterized by a higher T3 concentration with no variation in the other thyroid hormones [25]. Till date, no conclusive data has been obtained for the seasonal variations of TSH and TH in a temperate climate; the maximum summer temperature has also not exceeded 35°C. The present study was performed in Sulaymaniyah city, situated 884.8 m above sea level having latitude 35° 34′ 0.7104^″^ north and longitude 45° 24′ 57.9852^″^ east. This place, with a significant difference between the climatic conditions in the summer and winter seasons, is the best region for studying the effect of climatic variations on the hormone level. In our study, we observed steady TSH and TH values in euthyroid and SCH subjects during summer and winter, except for a small but significant increase in FT3 in euthyroid individuals (*P* < 0.0001) and a small decrease in FT4 in SCH individuals (*P* < 0.05) during winter. The effect of duration of outdoor stay was only observed during winter in the FT3 level of euthyroid individuals, apart from which no appreciable change in pituitary-thyroid axis (PTA) function was observed in both the groups. We also found that the variations in the climatic components were associated with the TSH and TH levels in euthyroid subjects, with no effect on SCH subjects.

The small increase in FT3 in euthyroid subjects during winter was in agreement with previous studies carried out in temperate climates [16, 26]. In another study performed on 13 healthy male and 13 healthy female subjects in Belgium, the total T3 showed a lower value during spring and summer while TSH showed higher value during winter-spring [15]. In a study involving euthyroid and SCH subjects in Korea [19] the serum TSH increased during the winter and spring seasons leading to transition to SCH. And a study conducted for six years in Japan, another country with a temperate climate, reported a decreased TSH concentration during summer that got reversed in winter; there existed a negative correlation between the daily temperatures and TSH and FT3 concentrations [27]. But these two studies [19, 27] had a shortcoming; the entire study, i.e., the follow-up, was not carried out on the same subjects. The subjects who developed thyroid disease or were on thyroid treatment throughout the study could not be excluded because the studies were carried out retrospectively. In our study, no significant change in TSH was found indicating that the seasonal variations in the selected region had no appreciable effect on the diagnosis of SCH subjects, also in neither their monitoring nor treatment. The comparison of the climate components with the hormone levels of euthyroid subjects exhibited significant correlation with the thyroid hormone, FT3, and the FT3/FT4 ratio. This data suggests that the increase in FT3 and FT3/FT4 in winter is partly due to decreased temperature and sunshine and increased humidity, cloud cover, and atmospheric pressure, especially a day before sample collection. On the other hand, a decrease in TSH and FT4 was observed due to increased cloud cover and humidity, respectively. The study in Italy [16] focused only on the evaluation of the correlation between climatic components and TSH and found no significant correlation between them in euthyroid subjects while the study conducted in Belgium [15] focused on the association between climatic components and T3, and showed an inverse relationship. The negative correlation between temperature and FT3 level is in contrast to studies carried out in arctic or subarctic regions [6] which may partly be due to the difference in the latitude and partly due to the sample size and lesser participation when the study was conducted in subarctic region. The altitude of the region plays an important role in the outcome of the study. So far, the data obtained from both the cold and temperate climates have not been very conclusive as most of the studies have been done at high altitudes, where along with the temperature, factors such as hypobaric condition, changes in oxygen partial pressure, and increased ultraviolet radiation might affect the secretion of hormones. A study conducted in a hypobaric chamber demonstrated that a simulated altitude of 3810 m affects the TH level [28]. The studies are also influenced by the sleep pattern, dietary changes, and plasma volume shifts [29].

There are no comparative study between SCH and Euthyroid subjects correlating climatic components and hyroid hormone levels. Occurrence of subclinical hypothyroidism has been reported in 4% to 18% of the adult population [30], detected by mild elevation in TSH (≤10.0 mIU/l) and in most cases, SCH individuals revert to euthyroidism without treatment [31]. Subclinical hypothyroidism has been known to be influenced by age, sex, BMI, dietary iodine intake, ethnicity, smoking status, latent autoimmune thyroid diseases, and TSH secretion [18]. The basal TSH was found to be negatively correlated with the seasonal alterations in ambient temperature in a small number of hypothyroid patients on thyroxine treatment [19]. In addition, in primary hypothyroid patients, a small but significantly lower level of TH was found in winter suggesting that the dose required for the replacement of thyroid hormone in patients with hypothyroidism may be higher in winter than in summer [30].

Previous studies have shown that there can be more than one reason behind the response of hormone levels to seasonal variations. An increase in thyroidal secretion cannot be the only reason for the seasonal variation in TH level. In such a case, there should be an increase in both T3 and T4, but in most studies, T3 increased with no change or slight decrease in T4 in winter as mentioned earlier. It can be a primary response of the PTA axis to changing the light and ambient temperature [8], or it is due to the small decrease in either the volume of distribution or serum T4 [8, 19]. It is possible that seasonal changes in TH level are due to change in their metabolism [32] or the combination of these factors.

The prevalence of subclinical hypothyroidism is dependent on age, sex, and iodine intake, the prevalence being more in adults. Studies carried out in temperate climates have demonstrated that the prevalence of SCH is population specific, i.e., occurring both in iodine-sufficient countries [33] and in iodine-deficient populations [34, 35]. Reportedly, people with SCH and TSH levels exhibited higher measures of BMI and waist-to-hip ratio than subjects with normal or lower TSH levels [36]. Similarly, we also observed an increased TSH level in the SCH population who exhibited a higher BMI and waist circumference compared to the euthyroid population. Regular monitoring of SCH patients is necessary before the commencement of L-T4 replacement therapy as the condition can either progress to hypothyroidism or regress to normal thyroid function [37]. Thyroid hormone plays a major role in adaptive thermogenesis, an important target for T3 [38]. Mitochondrial uncoupling protein (UCP3) in skeletal muscle and the brown adipose tissue (BAT) is significantly related to cold-induced adaptive thermogenesis by the dissipation of heat due to uncoupling of the respiratory chain from oxidative phosphorylation [39]. The UCP3 is induced by T3 in addition to fatty acid [40]. The contribution of muscle is responsible on an average for 40% of thermogenesis in humans [38]. Most of the T3 in plasma comes from the conversion of T4 by extrathyroidal D2. The human study revealed that D2 is present in the skeletal muscle which has a role in the extrathyroidal T3 production. Cold exposure increases the sympathetic nervous system activity and increases the potential for cold tolerance by nonshivering thermogenesis in humans [41, 42]. It was previously thought that BAT is functional only in rodents and newborns [30], but recently functional BAT was discovered in an adult human, the primary location being the supraclavicular area of the body, and the uncoupling protein present was UCP1 [43].

Studies on the BAT, skeletal muscle, and thermogenesis are suggestive of the increase in D2 activity by cold exposure; this is in concordance with the present study. Based on the correlations found in the present study, we can suggest that the slight increase of FT3 and FT3/FT4 and the decrease of FT4 in winter are partly due to the effect of climate, especially one day before the sample collection, on the peripheral conversion of FT4 to FT3 and thus on D2 activity. Thus, we can conclude indirectly that lower temperature and sunshine duration, higher humidity, cloud cover, and atmospheric pressure present during a winter month can slightly increase the activity of D2 in peripheral tissue. Increased sunshine duration and temperature during summer decrease the activity of this enzyme slightly.

## 5. Conclusions

In conclusion, each climatic component such as humidity, sunshine duration, temperature, cloud cover, and atmospheric pressure has its share on the slight variance of hormone levels found in different seasons, the effect being mainly on FT3 and FT3/FT4 ratio. The effect of climate was mostly on peripheral metabolism and most probably on the conversion of FT4 to FT3 rather than central causes (PTA). The duration of outdoor exposure slightly affects hormone level, but no significant correlation exists between them. Since SCH had no significant difference in serum TSH apart from slightly higher FT4 during the summer season, it was concluded that seasonal variations in this temperate climate have no effect on the diagnosis of SCH cases. The data obtained for the SCH cases can be further confirmed by taking a large number of SCH subjects as a small sample size which does not give statistically significant results. The urinary T3 should have been measured to get a clear idea regarding the change in the rate of clearance of T3.

We would like to recommend a midlatitude study on the effect of the season and correlation of climatic components on the activity of D2 and UCP in BAT and skeletal muscle to check if decreased temperature and sunshine duration during winter can cause an increase in the activity of extrathyroidal D2 or BAT in humans.

## Figures and Tables

**Table 1:** Mean climate characteristics in summer and winter.

Mean climate components	Summer (mean ± SD)	Winter (mean ± SD)
Maximum temperature (°C)	41.67 ± 2.61	12.09 ± 4.05
Minimum temperature (°C)	26.5 ± 2.63	2.5 ± 2.56
Average temperature (°C)	34.11 ± 2.32	7.28 ± 2.58
Average humidity (%)	25.53 ± 3.57	66.85 ± 11.63
Cloud cover (okta)	0.083 ± 0.129	0.408 ± 0.311
Sunshine duration (hours)	10.76 ± 1.34	4.87 ± 3.14
Atmospheric pressure (mmHg)	1005.73 ± 12.56	1024 ± 3.65

Independent *t*-test used to find the difference between the summer and winter climate components. *P* < 0.001 in all components.

**Table 2:** Demographic characteristics of the study subjects.

Characteristics	Total *n* = 177 Mean ± SD *n* (%)	Euthyroid (Eu) *n* = 152 Mean ± SD *n* (%)	Subclinical hypothyroid (SCH) *n* = 25^∗^ Mean ± SD *n* (%)
Age	34 ± 12.46	34.24 ± 12.05	36.44 ± 14.82

Gender			
Male	39 (22)	37 (24.3)	2 (8)
Female	138 (78)	115 (75.7)	23 (92)

BMI	26.91 ± 4.58	26.78 ± 4.57	27.69 ± 4.63
Waist circumference (cm)	85.36 ± 12.03	84.64 ± 11.27	89.48 ± 15.40
TPO value (IU/ml)	33.25 ± 85.70	22.64 ± 53.54	97.76 ± 175.45

TPO positivity			
Negative	148 (84.1)	133 (88.1)	15 (60)
Positive	28 (15.9)	18 (11.9)	10 (40)

Season			
Summer	176	136 (77.3)	40 (22.7)
Winter	176	140 (79.5)	36 (20.5)

^∗^This number is based on those who are subclinical hypothyroid at summer and winter seasons; the subjects who are euthyroid at either season were excluded.

**Table 3:** Comparison of the serum TSH, FT4, FT3, and FT3/FT4 ratio between summer and winter season.

	Total	*P* value	EU	*P* value	SCH	*P* value
TSH (mIU/l)						
Summer	2.93 ± 1.96	0.136	2.34 ± 1.25	0.382	6.64 ± 1.60	0.171
Winter	2.77 ± 1.79		2.25 ± 1.25		6.04 ± 1.17	

FT4 (ng/dl)						
Summer	1.23 ± 0.57	*0.007*	1.23 ± 0.15	0.063	1.21 ± 0.15	*0.009*
Winter	1.20 ± 0.16		1.21 ± 0.16		1.14 ± 0.14	

FT3 (pg/ml)						
Summer	3.23 ± 0.44	*<0.001*	3.22 ± 0.44	*<0.001*	3.27 ± 0.45	0.853
Winter	3.37 ± 0.39		3.39 ± 0.40		3.25 ± 0.32	

FT3/FT4 ratio						
Summer	2.68 ± 0.45	*<0.001*	2.65 ± 0.45	*<0.001*	2.81 ± 0.44	*0.037*
Winter	2.87 ± 0.43		2.84 ± 0.43		2.99 ± 0.43	

The parameters are expressed as the mean ± SD, and the means are compared using a Paired sample *t*-test.

**Table 4:** Comparison of the serum TSH, FT4, and FT3 between degrees of exposure to environmental temperature in summer and winter seasons.

Parameters	Outdoor stay hour		
	Slight	Good	
TSH (mIU/l)			
Summer	3.02 ± 2.01	2.72 ± 1.81	0.385
Winter	2.86 ± 1.91	2.52 ± 1.41	0.275

FT4 (ng/dl)			
Summer	1.23 ± 0.15	1.22 ± 0.15	0.858
Winter	1.19 ± 0.16	1.21 ± 0.15	0.491

FT3 (pg/ml)			
Summer	3.18 ± 0.42	3.30 ± 0.46	0.191
Winter	3.30 ± 0.34	3.49 ± 0.45	*0.023*

FT3/FT4 ratio			
Summer	2.64 ± 0.442	2.76 ± 0.45	0.196
Winter	2.84 ± 0.44	2.94 ± 0.35	0.275

The parameters are expressed as the mean ± SD, and the means are compared using a Paired sample *t*-test.

**Table 5:** Correlations of climate parameters of one day, one week, and one month before blood sample collection with the mean TSH, FT3, FT4, and FT3/FT4 ratio.

Duration from sample taking	Euthyroid				
	Climate parameters	FT3 (pg/ml)	FT4 (ng/dl)	FT3/FT4 ratio	TSH (mIU/l)
One day	Temperature	*-0.195* ^∗^	0.029	*-0.193* ^∗^	0.027
	Humidity	*0.204* ^∗^	-0.032	*0.222* ^∗^	-0.046
	Sunshine duration (hours)	*-0.203* ^∗^	0.098	*-0.264* ^∗∗^	0.077
	Cloud cover (octa)	0.140	*-0.136* ^∗^	*0.254* ^∗∗^	-0.085
	Atmospheric pressure	*0.174* ^∗^	-0.02	0.151	-0.001

One week	Temperature	*-0.220* ^∗∗^	0.058	-0.239	0.007
	Humidity	*0.227* ^∗∗^	-0.050	*0.212* ^∗∗^	-0.033
	Sunshine duration (hours)	-0.141	0.033	-0.142	0.07
	Cloud cover (octa)	-0.041	0.001	-0.042	-0.021
	Atmospheric pressure	0.067	-0.004	0.057	-0.014

One month	Temperature	-0.143	0.045	*-0.172* ^∗^	0.003
	Humidity	0.124	-0.02	0.136	-0.037
	Sunshine duration (hours)	-0.127	0.05	*-0.202* ^∗^	0.023
	Cloud cover (octa)	0.108	-0.021	*0.174* ^∗^	-0.005
	Atmospheric pressure	*0.168* ^∗^	-0.035	*0.186* ^∗^	0.001

Correlations between parameters were found using Pearson's correlation. ^∗^*P* value < 0.05 and ^∗∗^*P* value < 0.01.

## Data Availability

The data is available in public at the following links: https://figshare.com/articles/slemany_x2017_xlsx/6796184, https://figshare.com/articles/Climate_data_of_Sulaimaniyah_city/6796193, and https://figshare.com/articles/study_sample_data/6804089.

## References

[1] Palmer K. (2015). Disorders of the thyroid gland-endocrinology and metabolism. *MKSAP 17*.

[2] Maia A. L., Kim B. W., Huang S. A., Harney J. W., Larsen P. R. (2005). Type 2 iodothyronine deiodinase is the major source of plasma T_3_ in euthyroid humans. *Journal of Clinical Investigation*.

[3] Hall J. (2015). Thyroid metabolic hormones synthesis and secretion of the thyroid metabolic hormones. *Guyton and Hall Textbook of Medical Physiology*.

[4] Ortiga-Carvalho T. M., Chiamolera M. I., Pazos-Moura C. C., Wondisford F. E. (2016). Hypothalamus-pituitary-thyroid axis. *Comprehensive Physiology*.

[5] Palinkas L. A., Reedy K. R., Shepanek M. (2007). Environmental influences on hypothalamic–pituitary–thyroid function and behavior in Antarctica. *Physiology & Behavior*.

[6] Hassi J., Sikkilä K., Ruokonen A., Leppäluoto J. (2001). The pituitary – thyroid axis in healthy men living under subarctic climatological conditions. *The Journal of Endocrinology*.

[7] Do N. V., Mino L., Merriam G. R. (2004). Elevation in serum thyroglobulin during prolonged Antarctic residence : effect of thyroxine supplement in the polar 3, 5, 3-triiodothyronine syndrome. *The Journal of Clinical Endocrinology and Metabolism*.

[8] Palinkas L. A., Reed H. L., Reedy K. R., Do N. V., Case H. S., Finney N. S. (2001). Circannual pattern of hypothalamic–pituitary–thyroid (HPT) function and mood during extended antarctic residence. *Psychoneuroendocrinology*.

[9] Sarne D. (2016). *Effects of the Environment, Chemicals and Drugs on Thyroid Function*.

[10] Levine M., Duffy L., Moore D. C., Matej L. A. (1995). Acclimation of a non-indigenous sub-Arctic population: seasonal variation in thyroid function in interior Alaska. *Comparative Biochemistry and Physiology. Part A, Physiology*.

[11] Haus E. (2007). Chronobiology in the endocrine system. *Advanced Drug Delivery Reviews*.

[12] Reed H. L., Reedy K. R., Palinkas L. A. (2001). Impairment in cognitive and exercise performance during prolonged Antarctic residence: effect of thyroxine supplementation in the polar triiodothyronine syndrome 1. *The Journal of Clinical Endocrinology and Metabolism*.

[13] Qi X., Chan W. L., Read R. J., Zhou A., Carrell R. W. (2014). Temperature- responsive release of thyroxine and its environmental adaptation in Australians. *Proceedings of the Royal Society B: Biological Sciences*.

[14] Andersen S., Bruun N. H., Pedersen K. M., Laurberg P. (2003). Biologic variation is important for interpretation of thyroid function tests. *Thyroid*.

[15] Maes M., Mommen K., Hendrickx D. (1997). Components of biological variation, including seasonality, in blood concentrations of TSH, TT3, FT4, PRL, cortisol and testosterone in healthy volunteers. *Clinical Endocrinology*.

[16] Gullo D., Latina A., Frasca F., Squatrito S., Belfiore A., Vigneri R. (2017). Seasonal variations in TSH serum levels in athyreotic patients under L-thyroxine replacement monotherapy. *Clinical Endocrinology*.

[17] Smals A. G. H., Ross H. A., Kloppenborg P. W. C. (1977). Seasonal variation in serum T3 and T4 levels in man. *The Journal of Clinical Endocrinology and Metabolism*.

[18] Simoni M., Velardo A., Montanini V., Fustini F., Seghedoni S., Manama P. (1990). Circannual rhythm of plasma thyrotropin in middle-aged and old euthyroid subjects. *Hormone Research*.

[19] Kim T. H., Kim K. W., Ahn H. Y. (2013). Effect of seasonal changes on the transition between subclinical hypothyroid and euthyroid status. *The Journal of Clinical Endocrinology and Metabolism*.

[20] Karmisholt J., Andersen S., Laurberg P. (2008). Variation in thyroid function tests in patients with stable untreated subclinical hypothyroidism. *Thyroid*.

[21] Chen N., Wu Q., Li H., Zhang T., Xu C. (2016). Different adaptations of Chinese winter-over expeditioners during prolonged Antarctic and sub-Antarctic residence. *International Journal of Biometeorology*.

[22] Palinkas L. A., Reedy K. R., Shepanek M. (2010). A randomized placebo-controlled clinical trial of the effectiveness of thyroxine and triiodothyronine and short-term exposure to bright light in prevention of decrements in cognitive performance and mood during prolonged Antarctic residence. *Clinical Endocrinology*.

[23] Mordes J. P., Blume F. D., Boyer S., Zheng M.-R., Braverman L. E. (1983). High-altitude pituitary–thyroid dysfunction on Mount Everest. *The New England Journal of Medicine*.

[24] Basu M., Pal K., Malhotra A. S., Prasad R., Sawhney R. C. (1995). Free and total thyroid hormones in humans at extreme altitude. *International Journal of Biometeorology*.

[25] Savourey G., Garcia N., Caravel J. P. (1998). Pre-adaptation, adaptation and de-adaptation to high altitude in humans: hormonal and biochemical changes at sea level. *European Journal of Applied Physiology and Occupational Physiology*.

[26] Koono N. (1980). Reciprocal changes in serum concentrations of triiodothyronine and reverse triiodothyronine between summer and winter in normal adult men. *Endocrinologia Japonica*.

[27] Yoshihara A., Noh J. Y., Watanabe N. (2018). Seasonal changes in serum thyrotropin concentrations observed from big data obtained during six consecutive years from 2010 to 2015 at a single hospital in Japan. *Thyroid*.

[28] Rawal S. B., Singh M. V., Tyagi A. K., Chaudhuri B. N. (1993). Thyroidal handling of radioiodine in sea level residents exposed to hypobaric hypoxia. *European Journal of Nuclear Medicine*.

[29] Reed H. (2005). Environmental influences upon thyroid hormone regulation. *Werner and Ingbar’s the Thyroid*.

[30] Villar H. C. C. E., Saconato H., Valente O., Atallah Á. N., Cochrane Metabolic and Endocrine Disorders Group (2007). Thyroid hormone replacement for subclinical hypothyroidism. *Cochrane Database of Systematic Reviews*.

[31] Meyerovitch J., Rotman-Pikielny P., Sherf M., Battat E., Levy Y., Surks M. I. (2007). Serum thyrotropin measurements in the community. *Archives of Internal Medicine*.

[32] Fisher D. A. (1996). Physiological variations in thyroid hormones: physiological and pathophysiological considerations. *Clinical Chemistry*.

[33] Teng W., Shan Z., Teng X. (2006). Effect of iodine intake on thyroid diseases in China. *The New England Journal of Medicine*.

[34] Guan H., Shan Z., Teng X. (2008). Influence of iodine on the reference interval of TSH and the optimal interval of TSH: results of a follow-up study in areas with different iodine intakes. *Clinical Endocrinology*.

[35] Laurberg P., Pedersen K. M., Hreidarsson A., Sigfusson N., Iversen E., Knudsen P. R. (1998). Iodine intake and the pattern of thyroid disorders: a comparative epidemiological study of thyroid abnormalities in the elderly in Iceland and in Jutland, Denmark. *The Journal of Clinical Endocrinology and Metabolism*.

[36] Wang J., Ma X., Qu S. (2013). High prevalence of subclinical thyroid dysfunction and the relationship between thyrotropin levels and cardiovascular risk factors in residents of the coastal area of China. *Experimental and Clinical Cardiology*.

[37] Karmisholt J., Andersen S., Laurberg P. (2011). Variation in thyroid function in subclinical hypothyroidism: importance of clinical follow-up and therapy. *European Journal of Endocrinology*.

[38] Vybíral S., Lesná I., Jansky L., Zeman V. (2000). Thermoregulation in winter swimmers and physiological significance of human catecholamine thermogenesis. *Experimental Physiology*.

[39] Muzzin P., Boss O., Giacobino J. P. (1999). Uncoupling. Protein 3: its possible biological role and mode of regulation in rodents and humans. *Journal of Bioenergetics and Biomembranes*.

[40] Lanni A., Moreno M., Lombardi A., Goglia F. (2003). Thyroid hormone and uncoupling proteins. *FEBS Letters*.

[41] Mooventhan A., Nivethitha L. (2014). Scientific evidence-based effects of hydrotherapy on various systems of the body. *North American Journal of Medical Sciences*.

[42] Esler M., Rumantir M., Wiesner G., Kaye D., Hastings J., Lambert G. (2001). *Sympathetic Nervous System and Insulin Resistance: From Obesity to Diabetes*.

[43] van Marken Lichtenbelt W. D., Vanhommerig J. W., Smulders N. M. (2009). Cold-activated brown adipose tissue in healthy men. *The New England Journal of Medicine*.

